# Mindfulness in Politics: A Qualitative Study on Mindfulness Training in the UK Parliament

**DOI:** 10.1007/s12671-023-02156-x

**Published:** 2023-05-29

**Authors:** Otto Simonsson, Christine Bergljottsdotter, Jayanth Narayanan, Stephen Fisher, Jamie Bristow, Ruth Ormston, Richard Chambers

**Affiliations:** 1 Department of Sociology, University of Oxford, Oxford, UK; 2 Department of Neurobiology, Care Sciences and Society, Karolinska Institute, Stockholm, Sweden; 3 Department of Work- and Organizational Psychology, Stockholm University, Stockholm, Sweden; 4 NUS Business School, National University of Singapore, Singapore, Singapore; 5 The Mindfulness Initiative, London, UK; 6 Monash Centre for Consciousness & Contemplative Studies, Monash University, Melbourne, Australia

**Keywords:** Empathy, Compassion, Leadership, Meditation, Mindfulness, Politics

## Abstract

**Objectives:**

While mindfulness in the workplace has received substantial scientific attention in the past decades, it is not yet well-understood if, under what circumstances, and in what ways mindfulness training may be helpful for individuals working in political environments. The aim of this study was to explore the experience of mindfulness training among British politicians, as well as mindfulness facilitators who had taught mindfulness to politicians in the UK Parliament.

**Method:**

Between September and November 2021, semi-structured in-depth interviews were conducted with British politicians (*n* = 18) who had experience of mindfulness training and mindfulness facilitators (*n* = 4) who had taught mindfulness to politicians in the UK Parliament. The interview material was analyzed using reflexive thematic analysis.

**Results:**

Two main themes related to the experience of mindfulness training in politics were developed during the analytic process: (1) mindfulness approaches addressing particular challenges in political work, and (2) mindfulness sessions and group dynamics. Taken together, mindfulness training helped the politicians to better deal with the demands and stresses of political work, to reconnect with themselves and be more grounded, and – especially when taught in a group setting – to relate to other politicians and their viewpoints in a more humane and constructive way.

**Conclusions:**

The results suggest that mindfulness training can be beneficial at both the personal and professional level for individuals working in political contexts, which can provide a rationale for governments to introduce mindfulness-based programs to politicians.

**Supplementary Information:**

The online version contains supplementary material available at 10.1007/s12671-023-02156-x.

Mindfulness training has become increasingly widespread in British society, with 15% of adults in Britain in 2018 estimated to have learnt to practice mindfulness (Simonsson et al., 2021). There are now mindfulness-based programs in a range of settings, including the education system, the workplace, and the military (Creswell, 2017). Notably, mindfulness training has also been introduced in the UK Parliament (Bristow, 2019), where hundreds of politicians from different political parties have attended a mindfulness program adapted from Mindfulness-Based Cognitive Therapy (MBCT; Segal et al., 2018): the Finding Peace in a Frantic World curriculum (Williams & Penman, 2011).

The introduction of mindfulness training in the UK Parliament has been accompanied by books and research on mindfulness in politics (Alkoby et al., 2017; Cook, 2016; Ferguson, 2016; Kabat-Zinn, 2005; Leggett, 2022; McLeod, 2006; Petersen & Mitkidis, 2019; Purser, 2019; Ramstetter, 2021; Ryan, 2012). For instance, one study (Simonsson et al., 2022a) randomized participants who identified with either the Remain side (i.e., Remainers) or the Leave side (i.e., Leavers) of the Brexit debate into an 8-week mindfulness course (based on the Finding Peace in a Frantic World curriculum) or a waitlist control condition. The findings showed that affective polarization – that is, the difference in feelings toward the political ingroup and outgroup (Iyengar et al., 2019) – was reduced for participants in the mindfulness condition, relative to participants in the waitlist control condition. Another randomized controlled trial with a sample of Remainers and Leavers found that participants randomly allocated to listen to a brief befriending meditation (an exercise used in the Finding Peace in a Frantic World curriculum that cultivates friendship and kindness toward oneself and others) scored lower on affective polarization than participants randomly allocated to listen to an active control condition (Simonsson et al., 2022b; see also Simonsson et al., 2022c). Taken together, the findings in these studies suggest that mindfulness meditation and other related exercises may be effective tools to reduce affective polarization, but there may also be other ways in which such exercises could be useful in politics.

In a review article, Bristow (2019) described the developments of mindfulness in politics and identified potential benefits of mindfulness training for elected officials: attention, impulse control, kindness toward self and others, and meta-cognition. These suggested benefits broadly mirror personal benefits described by the former Labour Party Member of Parliament (MP) Chris Ruane, who credits mindfulness training with changes in his relationship to himself, others, and the world at large (Ruane, 2021). Given the limited qualitative research on mindfulness in politics, however, it is important to build on these accounts with qualitative scientific studies that can capture the human potential of mindfulness training and provide a better understanding of whether, under what circumstances, and in what ways mindfulness meditation and other related exercises may be helpful for individuals working in political environments. Hence, the aim of the present study was to explore the experience of mindfulness training in politics. Using a sample of 22 participants, we conducted semi-structured, in-depth interviews with 18 British politicians who had experience of mindfulness training and four mindfulness facilitators who had taught mindfulness to politicians in the UK Parliament.

## Method

### Participants

The participants in this study comprised a convenience sample of British politicians who had experience of mindfulness training and mindfulness facilitators who had taught mindfulness to politicians in the UK Parliament. The British politicians were interviewed to provide a first-person perspective on mindfulness training for individuals working in political environments. The mindfulness facilitators were interviewed to provide both a first-person perspective on the experience of teaching mindfulness to individuals working in political environments and also a third-person perspective on the experience of the politicians. The participants were recruited via email through the Mindfulness Initiative, which is a policy institute working with politicians who practice mindfulness. In total, 66 politicians and 4 mindfulness facilitators who were deemed likely to agree to an interview were sent emails with information about the study and an invitation to participate in an interview. Those who accepted the request were subsequently interviewed by the first author of the present study (OS).

Between September and November 2021, semi-structured, in-depth interviews were conducted with 18 politicians and four mindfulness facilitators. The group of politicians was relatively heterogenous with regard to age (mean age at the time of interview = 64 years, age range = 44–83), gender (10 females, 8 males), political party (Labour Party = 10, Conservative Party = 4, other political parties = 4), and current or most recent chamber affiliation (House of Lords = 10, House of Commons = 8). The 4 mindfulness facilitators (2 females, 2 males) had a mean age of 55 at the time of the interview (range = 52–59) and had extensive personal experience of mindfulness training and also extensive experience with teaching mindfulness.

### Procedure

The interviews took place via Zoom (https://zoom.us/) and participants provided verbal consent before the interviews were started. The interviews lasted between 15 and 60 min, were audio recorded, transcribed verbatim, analyzed, and then erased. The interviews were based on a semi-structured guide (Busetto et al., 2020) which covered the spectrum of interest with pre-defined, open-ended questions, exploring the ways in which participants experienced mindfulness training in politics.

### Mindfulness Program

From its inception in early 2013 until early 2020 when the COVID-19 pandemic precluded physical attendance, the mindfulness program in the British Parliament entailed two to three introductory courses (based on the Finding Peace in a Frantic World curriculum) per year, comprising of eight weekly sessions of 75 min each. There was also an hour-long guided practice group once per week for those who had already attended a course. The sizes of training cohorts ranged from 5 to 25 participants. By 2020, 350 politicians had taken some part in an introductory course. Between 2018 and 2020, some politicians and their spouses also attended a number of silent practice days held on Sundays close to the parliamentary estate. Delivered in parallel to the courses for politicians, 800 members of their staff have also received similar training since 2013. The progam has recently been opened to all staff on the parliamentary estate.

Following the outbreak of the COVID-19 pandemic, there was a hiatus until courses and drop-in groups started to be held online. The program was redesigned to involve shorter 6-week introductory courses with 60-min sessions that feature more neuroscience and workplace-specific content. There was also an increase to two drop-in groups per week.

### Data Analyses

Reflexive thematic analysis was used to identify and make sense of patterns of meaning across the data set (Braun & Clarke, 2006, 2012, 2020). This approach was chosen to ensure that the data was collected and analyzed in such a way that it both respected and expressed the subjectivity of the participants’ experiences, while also acknowledging the reflexive influence of the researchers’ interpretations (Byrne, 2022). Epistemologically, a social constructionist approach (i.e., meaning is co-constructed between researchers and participants; Ng et al., 2019) was used. An experiential orientation was also used since the aim of the study was to focus on participants’ own accounts of their experiences of mindfulness training in politics. A predominantly inductive (or *data-driven*) approach was adopted which emphasised respondent-based meanings and open coding of data (i.e., codes solely reflect content of the data and remain free from any conceptual framework or pre-conceived theory; Clarke & Braun, 2013). Deductive (or *theory-driven*) analysis was also employed to a small degree in order to ensure that the open-coding contributed to the development of themes that were meaningful to the research questions. It is common within reflexive thematic analysis to use a combination of inductive and deductive approaches (Braun & Clarke, 2019, 2020; Clarke & Braun, 2013), although one of them usually tend to dominate over the other (Braun & Clarke, 2012). Both semantic and latent coding were utilized (i.e., codes can be described as both a descriptive analysis of the data and the researchers’ attempts to identify hidden meanings or underlying assumptions that may inform the semantic content of the data). By adopting latent coding, the researchers played an active role in the interpretation of codes and themes while also identifying which were relevant to the research questions (Braun & Clarke, 2020).

The first two authors (OS and CB) engaged in the reflective thematic analytic process, which included six phases (as recommended by Braun & Clarke 2006, 2012, 2020; Campbell et al., 2021). The first step involved getting familiar with the interview material by reading, re-reading, and continually making notes on the content. Next, OS and CB coded the data independently, which generated a diversity of codes. The coding involved using a text document to highlight relevant text passages with a descriptive code. OS and CB then reviewed and discussed the codes together. Following the coding, OS and CB jointly engaged in the process of developing, testing and reviewing potential themes. This included identifying coherent patterns and reviewing the entire data set as a whole, collapsing overlapping themes into a single theme and ensuring that there was enough data to support each theme, as well as fitting the broader story of the data set in line with the research questions. The final step of writing everything up involved revisiting the research questions and reviewing the meaning within as well as between themes.

In order to ensure the quality of the study, transcripts were checked with audio recordings and researcher triangulation was employed. OS and CB coded the data independently, but they also discussed their impressions of the data regularly. These regular meetings ensured examination of the data from multiple perspectives and allowed the researchers’ interpretations to be challenged. The personal experience of mindfulness practice that OS and CB had was critically considered to ensure that their own perspectives did not bias the interpretation of the data.

## Results

Two main themes related to the experience of mindfulness training in politics were developed during the analytic process: (1) mindfulness approaches addressing particular challenges in political work and (2) mindfulness sessions and group dynamics (see Supplementary Information for additional theme related to work environment challenges in political culture). Each theme is presented below and exemplified with illustrative quotes.

### Mindfulness Approaches Addressing Particular Challenges in Political work

#### Politicians’ Perspective

A majority of the politicians indicated that the mindfulness training helped them to address a range of challenges inherent in their daily political work. For example, the mindfulness training provided them with the tools to deal with – and to recover from – stressful work situations.

The weekly classes are like oases in the desert, they are crucial to me. I’m like a drunk man going along the street clutching at lampposts. And the weekly mindfulness classes are those lampposts… It’s that moment of calm, of rebasing, of regrouping, of reconnecting, of rediscovering perspective and proportion, that is so important. Politician #9.

I just go away and sit in the library and do a few mindfulness practices. Sometimes just doing a short meditation, a body scan, or things like counting backwards and forwards – something that gets me away from the ruling program of meetings, speaking in debates, that kind of thing. Politician #3.

Ten minutes of [mindfulness] practice and all of a sudden you’re in a much better place. Politician #15.

The politicians also employed different mindfulness exercises in connection to various situations to manage the challenges in daily work more constructively. There were many of them, for instance, who described using mindfulness exercises to ground themselves before work meetings or even just before giving a speech.

[Before a speech] I would do the kind of grounding just to have a few moments – the breathing, the settling, to be aware of how I’m standing or sitting… I think it’s just good to be calm and grounded before a speech. Politician #18.

I think, to be honest, sometimes I do get sort of nervous before a speech – depends on the occasion. And it is very helpful to kind of just take a deep breath before you kind of jump up and have your say. Politician #11.

The mindfulness exercises made it possible for the politicians, at least momentarily, to connect with themselves and sense how they were feeling, get a renewed perspective on what was important in that moment, and continue work with a sense of balance. It increased their ability to relax, understand and manage negative thoughts and feelings, prioritize among their tasks and duties, and be efficient at work.

I’m better and more robust and able to move between things faster… [mindfulness] helps me do my job better… I’m more relaxed, I’m more focused. I’m more able to do a higher volume of work. I’m more able to move between things more easily and I’m less reliant on other methods to relax, which might involve alcohol. Politician #5.

And in mindfulness, basically, what you do is somehow manage those pointless negative thoughts that get in the way of you doing anything properly or anything in the right sequence. Politician #13.

Notably, the mindfulness training was described by many of the politicians as particularly useful in situations characterized by differences of opinion on important or highly charged issues. Such situations would typically lead to heated debates and polarized discussions, but the mindfulness training made it possible to calm down for a moment and consider several alternative ways of responding, which at times included not responding at all (e.g., not sending an e-mail or saying something impulsively when feeling angry).

If I can stop for literally one second to think: ‘I need to process that.’ Mindfulness gives me the time to literally calm my body down, to find space, to think: ‘Okay, I’m not going to react immediately. What is that person saying to me and why are they saying it?’ Politician #6.

Responding instead of reacting. The difference between an immediate reaction and a response – half-a-second difference. Think about how many political careers that have ended because of that… It’s a gift to be able to put that half-a-second difference between an immediate reaction and a response… It’s serving me in my private relationships and in my public ones. Politician #2.

There was also a general sense among the politicians that the mindfulness training helped them to listen non-judgmentally and empathically to politicians with opposing opinions. This, in turn, created the possibility of engaging with other politicians in a respectful conversation while still disagreeing on specific issues.

I just think [mindfulness] makes me more considerate. As opposed to always being so stressed that you react, I’m much more able to step back and see the other point of view and let it pass if it’s something that maybe irritates me. Politician #18.

Mindfulness taught me a lot about other people’s states of mind… I think it makes any group of people more generous towards each other, more understanding, able to disagree, but also able to understand that when you disagree it doesn’t mean that you’re judging each other to be a worse person or an impossible person or whatever… Mindfulness connects you with other people and breaks down that sense of isolation. Politician #13.

I think [mindfulness] can be quite calming. When you’re mindful, it can just calm you down and so if you’re in this intense political arena, you can kind of calm the situation with mindfulness and you can even project that into the situation. Politician #16.

#### Mindfulness Facilitators’ Perspective

The mindfulness facilitators described how the mindfulness sessions were often competing with many other commitments that the politicians had, which usually meant that the politicians who attended the mindfulness sessions could be called away at the very last minute. This, in turn, made it difficult to maintain high levels of attendance, but those who did attend seemed to become less stressed and more grounded, more able to self-regulate their emotional reactions and responses, and more able to focus on the present and let go of negative thought patterns.

[The politicians] have a really challenging job and a very difficult [work] environment. It feels really helpful to be providing some support for the people who run our country to be as clear and grounded and present and awake as possible. And to be able to do their job and to flourish as human beings as well… At the end of the course, [the politicians say] that they feel more grounded, they feel more able to let go of negative thought patterns and anxiety, and [they feel more] focused on what is present. They feel they have tools to be calmer under stress and less reactive. Mindfulness Facilitator #2.

Another key challenge of teaching mindfulness to politicians was that the participants often belonged to different political parties, which made it challenging for the mindfulness facilitators to create a safe, warm, and friendly environment in which the participants could be vulnerable. The mindfulness facilitators therefore focused a lot on exercises that could bridge the political divides (e.g., befriending meditation, random acts of kindness) and create a sense of common humanity (i.e., a sense of all participants, irrespective of political affiliation, being part of the human race). Although these were some of the most counter-cultural exercises in the program, they seemed to play an important role in building trust and connectedness among the participants.

I did always teach the befriending meditation, [which] was the most counter-cultural [exercise]. And in the Finding Peace course, they´re invited to do random acts of kindness… And some of them would come back… saying [they] had the most amazing week of doing random acts of kindness for all kinds of people and really finding that powerful. Mindfulness Facilitator #3.

When talking about sensations in the feet and breathing and the wandering mind, [the politicians] realize they have these things in common. It doesn’t matter whether you’re Tory or Labour, or black or white, or young or old. Body sensations are body sensations, they don’t bring color or politics or age with them. It was lovely that you actually did sense that maybe they were able to relate to each other, in a way that they would never normally have the opportunity to do… And that’s a great thing about mindfulness, it appeals to our common humanity and that was really there in the room, which was lovely… I suppose you do feel like you have sowed seeds which have lasted in some of them. Mindfulness Facilitator #1.

### Mindfulness Sessions and Group Dynamics

#### Politicians’ Perspective

The mindfulness training appeared to lead to a shift in how the politicians viewed themselves and other politicians, which facilitated empathy and a sense of seeing each other as human beings beyond political divides.

[Mindfulness] is more important than ever when you’ve got a politics, certainly in this country, which is becoming ever more polarized, which is based on ever more fervent culture wars. And all of that really is based on attacking people based on their identities. And the more that we’ve got a little bit more of a gap between people’s identities, if you like, and beliefs they happen to have or thoughts they happen to think, then it feels like there might be a potential at least for politics based on greater respect and thoughtfulness. Politician #8.

I think mindfulness has helped to mitigate the narrowness and the bigotry of tribalism that distorts and hardens the political culture, in our country at any rate. We have a particularly adversarial political system because of our electoral system and we don’t have a political culture of compromise. And I’m not suggesting that mindfulness has led to a greater willingness to compromise, but it’s led to, I hope, a wiser perspective on the issues that divide us. Politician #9.

Certainly when I have political disagreements, [mindfulness] is so powerful in reminding me that I’m dealing with another human being and that we have different opinions. It doesn’t mean I have to dislike someone or be disliked… It helps to remove mental and psychological barriers that we place when we construct our enemies, when we construct our opposition. They’re not our enemies. They’re not our opposition. I’m not their opposition either. It’s just that, at that moment, we are in different positions and those positions will change because that is the only thing that is certain: change. Politician #10.

Notably, the very fact that the mindfulness sessions were delivered in a group setting that consisted of politicians from different political parties – who simply listened to others without the need to argue or defend their own position – seemed to create a sense of psychological safety and reduce animosity between politicians. It provided an opportunity for the politicians to leave their identity politics at the door and listen to other politicians in a safe setting, which facilitated bonding and brought the politicians closer to each other.

For politicians in particular, where we spend most of our time criticizing one another’s beliefs… Sharing the room with people whose political views I would not share… You see the human side and people were willing to make themselves quite vulnerable in terms of what was happening in their own lives… And I think that generates a particular sort of atmosphere of safety and mutual confidence. Politician #8.

Underneath all that nonsense going on, there are real people. You have the same pain, the same anxieties, the same worries, the same everything that we all have. And accepting them as people, as a person rather than their political ideology, I think mindfulness has helped me do that… [But] maybe it’s the group setting. Politician #12.

I care more about them. I care what happens to them, how they’re doing and how they’re managing. It is a very humanizing experience… I think essentially it was the group session because there you [meet] people from such different political positions and social positions and everything. Politician #13.

It’s brought everybody together. Somebody’s party politics is completely and utterly irrelevant when you’re sitting in the same room with an instructor doing the [mindfulness] course. I think it’s brought a better understanding and tolerance and engagement in people of different political persuasions… I think you’re more likely to be kinder to somebody who the previous evening you’ve done a mindfulness course [with and with whom] you come from the same understanding. Politician #7.

#### Mindfulness Facilitators’ Perspective

The group setting of the mindfulness sessions was perceived as both a challenge and an opportunity by the mindfulness facilitators. For instance, the participants were used to a parochial us-versus-them mindset and constantly criticizing politicans from other political parties. It meant that the mindfulness facilitators had to be sensitive about the fact that certain exercises (e.g., light yoga and stretching) might be perceived as exposing, at least before psychological safety had developed in the group. Once it had emerged, however, the openness and sharing of experiences within the group created a sense of togetherness that made the participants realize that politicians from different parties could get along – the divisions that characterized the political culture did not have to be there all the time.

I suppose you’re sensitive to their sensitivities about perceiving each other and opening their hearts to each other. In the end, that was very inspiring, actually, because what you realize is that these are people from different parties, different benches, and actually they would all get on. You could really create a sense of togetherness and team, which was lovely because you realize these divisions, these differences, they don’t have to be there all the time. It was quite inspiring actually at that level. But you were always just careful not to give them opportunities to be embarrassed or to reveal too much. Mindfulness Facilitator #1.

The mindfulness sessions were possibly the only context in which politicians could talk with their counterparts from other political parties about professional and personal challenges of being a politician. This seemed to create a strong sense of support and connectedness between politicians, regardless of their political affiliation.

The sense of the cross-party connections that they make in the class. That’s certainly something that I see that I find really valuable, that they support each other… There are not that many spaces, I think, where they can actually be human beings and be vulnerable and share their own challenges with others in that way. Mindfulness Facilitator #2.

I’m always amazed at the level of intimacy in the conversations. Let’s face it, these are people who are sitting on opposite sides of the room and usually in pretty adversarial conversations. And they sit and meditate together and talk about the challenges of COVID-19 or feeling more isolated… And as soon as you have connectedness, I think that really touches people. Mindfulness Facilitator #4.

## Discussion

This study explored the experience of mindfulness training in politics among British politicians, as well as mindfulness facilitators who had taught mindfulness to politicians in the UK Parliament. More specifically, we used reflexive thematic analysis to explore if, under what circumstances, and in what ways mindfulness training may be helpful for individuals working in political environments. The results indicated that the mindfulness training helped the politicians to better deal with the demands and stresses of political work, to reconnect with themselves and be more grounded, and – especially when taught in a group setting – to relate to other politicians and their viewpoints in a more humane and constructive way. These qualitative findings build on previous accounts of mindfulness training in the political arena (Bristow, 2019; Ruane, 2021) and results from prior quantitative studies on the effects of mindfulness training and other related exercises on affective polarization (Simonsson et al., 2022a, b, c).

The findings in this study broadly correspond with previous research on mindful leadership and mindfulness in the workplace (Donaldson-Feilder et al., 2019; Reb et al., 2014; Vonderlin et al., 2020). For example, studies on mindfulness in the workplace have shown reductions in stress and anxiety following a mindfulness intervention (Brendel et al., 2016; Schneider et al., 2010; Wasylkiw et al., 2015; see also Mellner et al., 2022). There is also evidence to suggest that mindfulness training can improve self-regulation, perspective-taking, and helping behavior among individuals in leadership positions (Urrila, 2022). These are valuable qualities for any leader, but it is especially important for elected officials – whose decision-making can impact millions of people – to invest time and resources into strengthening such qualities.

One particularly interesting finding in the present study was that the group setting of the mindfulness training appeared to have been helpful in cultivating a sense of connectedness among the British politicians in the mindfulness sessions, regardless of their political affiliation. Prior studies have compared mindfulness training delivered in group versus individually, but these studies have used different outcome measures and the results have been mixed (Mantzios & Giannou, 2014; Matiz et al., 2018; Schroevers et al., 2016). There have been criticisms on how mindfulness-based programs, in the way they are commonly delivered in organizations, may simply reinforce the individualized nature of contemporary working environments (Cook, 2016; Tomassini, 2016; Purser, 2019), which may, at least partially, be addressed by changing or adapting the content of the programs (e.g., integrating behavioral insights theory into the curriculum; see Lilley et al., 2022) and delivering the mindfulness training in a group setting, especially if the group consists of participants with different group identities.

The politicians who were interviewed in this study described a tribal and aggressive work environment culture in the UK Parliament, which pressured them into always appearing dominant, limited their ability to engage in rational debate, and fostered a culture of polarization (see Supplementary Information for additional theme related to work environment challenges in political culture). The Westminster model of government is a majoritarian system premised on adversarial debate between the government and the opposition. Other countries with proportional systems have more consensual politics with circular chambers (in contrast, the House of Commons has two sides facing each other) and more policy making in committees with deliberation and collaboration. While it is difficult to determine the extent to which the adversarial nature of British politics is a result of the parliamentary system in the country, the findings in this study suggest that mindfulness training can help politicians to have more constructive interactions with the people they work with and greater consideration of the circumstances of their adversaries. These are important steps toward promoting a more deliberative, constructive and consensual political culture, even within an institutional context that encourages adversarialism.

### Limitations and Future Research

This study included British politicians from different political parties and was carried out within the context of their day-to-day work activities, which provided a broad perspective on the phenomena under study (Graneheim & Lundman, 2004). The analysis was conducted by two researchers who, in a first step, analyzed the interview material independently from each other and, in a second step, discussed overlaps and divergences in their respective analyses to ensure that consensus was reached regarding the identified thematic categories. Although it is not expected within reflexive thematic analysis that codes and themes interpreted by one researcher are necessarily the same as those of another researcher, having multiple researchers sense-check ideas in a reflexive way and explore multiple interpretations of the data can contribute to a richer interpretation of meaning (Braun & Clarke, 2019).

Despite these strengths, it is important to consider limitations in this study when interpreting the results. First, this study used a convenience sample of British politicians and mindfulness facilitators who were deemed likely to agree to an interview. There are two types of selection bias that make it likely that the interviewed participants were biased toward being positive about the benefits of mindfulness: (1) choosing to participate in (or teach) any kind of mindfulness training in the first place and (2) being willing to participate in the study. Second, qualitative studies aim to provide in-depth understanding of a specific phenomenon in a certain population in a particular context and to identify the range of experiences and processes, even if it is not possible to determine anything about their generalizability (Carminati, 2018; Leung, 2015). The sampling may not have achieved theoretical saturation (Saunders et al., 2018), which would motivate further qualitative research on the topic. Third, although both the British politicians and the mindfulness facilitators were interviewed to provide first- and third-person perspectives on mindfulness training in politics (Frank & Marken, 2022), the qualitative nature of the study makes the findings susceptible to observer bias. Future studies could avoid this by exploring creative, quantitative research designs that can evaluate potential cause-and-effect relationships of mindfulness training in political contexts, including the effects on political discourse, political intergroup biases, and other outcomes related to politics.

In summary, the findings in the present study suggest that mindfulness training may have a range of personal and professional benefits for individuals working in political contexts. The delivery of the mindfulness training in a group setting, in particular, beyond being beneficial at the individual level, seems to have fostered more constructive interactions between – and greater consideration of the circumstances of – adversaries in a competitive context.

## Electronic Supplementary Material

Below is the link to the electronic supplementary material.

Supplementary Material 1

## Data Availability

The data cannot be shared openly with other researchers.
