# Supplementary material for: Mindfulness in Politics: A Qualitative Study on Mindfulness Training in the UK Parliament
Source: Mindfulness (N Y). 2023 May 29:1–9. Online ahead of print. doi: 10.1007/s12671-023-02156-x (PMC10225775; doi:10.1007/s12671-023-02156-x)
Supplement: Supplementary file 1 — Supplementary Material 1 [file 12671_2023_2156_MOESM1_ESM.docx]

**Supplementary Information**

**Journal: Mindfulness**

**Title: Mindfulness in politics: a qualitative study on mindfulness training in the UK Parliament**

**Authors:** Otto Simonsson^1,2^, Christine Bergljottsdotter (former Mellner)^3^, Jayanth Narayanan^4^, Stephen Fisher^1^, Jamie Bristow^5^, Ruth Ormston^5^, Richard Chambers^6^

^1^Department of Sociology, University of Oxford, Oxford, UK

^2^Department of Neurobiology, Care Sciences and Society, Karolinska Institute, Stockholm, Sweden

^3^Department of Work- and Organizational Psychology, Stockholm University, Stockholm, Sweden

^4^NUS Business School, National University of Singapore, Singapore, Singapore

^5^The Mindfulness Initiative, London, UK

^6^Monash Centre for Consciousness & Contemplative Studies, Monash University, Melbourne, Australia

Corresponding Author Contact Details

Otto Simonsson, otto.simonsson@ki.se

**Work environment challenges in political culture**

***Politicians’ perspective***

The work environment culture in the UK Parliament was described as combative and aggressive. The politicians described how members of opposing parties regularly attempted to undermine each other and take advantage of political weaknesses, which put pressure on politicians to always appear decisive and powerful. There was also a culture of strategically interrupting or disturbing political opponents to weaken their ability to engage in rational debate – a type of behavior that was perceived as bullying by many of the politicians.

It is very difficult to get through when you face people who sneer at you and call you names and have absolutely no sense of how to behave towards one another… It’s the anger behind all that and the fact that we don’t listen to each other. Politician #12

The bullying and abusive language was compounded by the sense of tribalism that defined the work environment in Westminster, where politicians tended to position themselves as members of one faction that was inherently antagonistic toward other factions. This, in turn, fostered a culture of judgment and polarization.

Other challenges within the political work environment included high workloads and time pressure. The politicians described running from one thing to another and trying to do too much, which resulted in high levels of stress. This was partly be explained by the fact that Members of the House of Lords do not generally have administrative support (e.g., assistants or secretaries), but there was also a fear of missing something or asking for help, in case it would be perceived by others as weakness.

It’s nonstop and people will fill your diary from eight o’clock in the morning to eight o’clock at night… Your day is chopped up into tiny little segments. By the end of the day, you feel sometimes quite exhilarated, but also very, very, very tired as well. It demands huge pressures. Politician #13

***Mindfulness facilitators’ perspective***

The mindfulness facilitators outlined work environment challenges that were largely congruent

with the descriptions of the politicians. They described a chaotic and frenetic work environment

in Westminster, where politicians try to work within a system that constantly asks more of them

than they can manage. It was therefore much more challenging to deliver mindfulness

training to politicians than other populations that the mindfulness facilitators had experience of

teaching mindfulness.

I think that they’ve got so many demands upon them that they rarely want to engage in [mindfulness]. They want that sense of space, of being able to be more effective. But they are so stimulated, they are so activated all the time. Mindfulness Facilitator #4

It’s really demanding – you feel like you get one chance with politicians. If they come to a class and they’re disappointed, they just won’t come back. The other things in their diaries will win. Mindfulness Facilitator #3
